# Informing the personalisation of interventions for parents of children with conduct problems: a qualitative study

**DOI:** 10.1186/s12888-020-02917-1

**Published:** 2020-10-20

**Authors:** Kathy McKay, Eilis Kennedy, Rob Senior, Stephen Scott, Jonathan Hill, Moira Doolan, Matt Woolgar, Siofra Peeren, Bridget Young

**Affiliations:** 1 University of Liverpool, Liverpool, UK; 2 Tavistock and Portman NHS Foundation Trust, 120 Belsize Lane, London, NW3 5BA UK; 3 Kings College, London, UK; 4 University of Reading, Reading, UK

**Keywords:** Children, Conduct disorder, Parenting programme, Personalisation, Mental health, Qualitative

## Abstract

**Background:**

Parenting programmes aim to alleviate behavioural problems in children, including conduct disorder. This study was part of a multi-phase mixed-methods project seeking to extend the reach of parenting programmes for the treatment of conduct problems through developing an evidence base to inform a personalised approach. It explored the narratives of parents of children with behavioural and conduct problems about parenting programmes to identify how such programmes could be personalised in order to extend their reach to parents and children who do not currently benefit.

**Methods:**

Face-to-face semi-structured interviews with a purposive sample of 42 parents, who had different experiences of parenting programmes. Interviews were conversational and informed by a topic guide. Analysis of transcripts of audio-recorded interviews drew on inductive thematic approaches and was framed largely within a phenomenological perspective.

**Results:**

Parents’ accounts demonstrated three themes: 1) a personalised approach needs to include the child; 2) a supportive school matters; and, 3) the programme needs to feel personal. Parents were more likely to have a positive experience at a parenting programme, and for their child to demonstrate positive behavioural changes, when they felt their concerns were validated within the group and they also felt supported by the child’s teachers. Parents whose children had been assessed prior to undertaking the programme were also more likely to perceive the programme to be beneficial, compared to parents who felt their child’s individual issues were never considered.

**Conclusions:**

Our findings point to the potential for personalised approaches to extend the reach of parenting programmes to parents and children who do not currently benefit from such programmes. Important in personalising parenting programmes is assessing children before parents are referred, to directly work with children as well as parents, and to work collaboratively with parents and children to identify which families are most suited to group support or one-to-one support and how this may change depending on circumstances.

## Background

Parenting programmes aim to alleviate behavioural problems in children. Programmes typically involve facilitator led group sessions to enhance a parent’s skills and confidence in managing their child’s behaviour, and the parent’s knowledge of child development and relationship with their child. There is strong evidence that group-based parenting programmes are effective in both younger [12, 22] and older children [6]. However, between 6% [20] and 40% of families do not benefit from these programmes including parents who drop out [10], and those who attend but whose children’s behaviour does not improve.

Evidence is conflicted on why drop-out rates are so varied across programmes, and so high for some programmes [14]. Drop-out has been linked to factors such socio-economic disadvantage, parental stress, the severity of the child’s behaviour, and combinations of these factors [2]. Systematic reviews of qualitative evidence have expanded on these findings. Continued engagement with a parenting programme is more likely when parents feel the group is ‘tailored’ for them personally (even if it’s not), where the group is made up of people with similar experiences, and where the facilitator engages with the parents outside of the sessions to support them as needed [1, 10]. Disengagement is more likely when parents do not feel the programme is helpful, where participation feels more of a burden, as well as when there are changes in family circumstances and a lack of accessibility [1, 10]. Moreover, the nature of the relationship between the facilitator and parent is important; with a strong therapeutic alliance shown to be associated with positive and sustainable changes in the parent [8].

While much of the focus has been on parenting programmes delivered to parents in face-to-face groups, survey research suggests that online parenting support can be useful [13]. There are also indications that online support improves accessibility for parents who would otherwise struggle to attend a face-to-face group [3]. Accessibility issues can also be resolved with one-on-one, home-based parenting programmes [15]. Further, parenting programmes have been adapted for teachers and implemented in school environments [18, 19].

In addition to the problems that programmes encounter in engaging parents, outcomes of programmes may vary for different subgroups of parents and children e.g., depressed mothers or children with callous unemotional traits [10]. Personalised approaches have therefore been proposed to enable intervention components to be tailored to the particular needs of such subgroups [9]. More recent evidence however from Individual Participant Data meta-analyses of IY parenting programmes suggest that sub-group differences may not be as significant as previously thought [6, 11, 12]. In mental health more broadly, personalisation has been conceptualised as interventions which privilege the *person,* their agency, and their interactions with the clinician, as factors that are intrinsic to the care process and outcomes [7].

## The Personalised Programmes for Children project

The study we report on here was part of the Personalised Programmes for Children (PPC) project. The PPC project is an ongoing multi-phase project where the early phases of PPC aimed to identify what aspects of parenting programmes might be personalised, and so inform the development of an intervention that will be evaluated in the later phases of PPC. The component that we report on here involved a qualitative interview study of parents’ narratives about Incredible Years (IY [23];, 2016), and other parenting programmes. We specifically aimed to interview parents who had different experiences of parenting programmes to inform the types of support that could help in engaging parents or carers who might otherwise not attend or drop out of parenting programmes, and for parents of children whose conduct does not improve despite parents engaging with a programme.

IY is “designed to promote social competence and prevent, reduce, and treat aggression and related conduct problems in young children (ages 3 to 10 years)” ([24], 32). It focuses on increasing positive parenting practices and decreasing negative parenting practices [20] and has been the subject of considerable research demonstrating its effectiveness [27].

This paper explores the narratives of parents of children with conduct problems about parenting programmes to identify how such programmes could be personalised in order to extend their reach to those who do not currently benefit.

## Methods

### Context

Early workstreams of PPC focused on the IY parenting programme delivered by a variety of service providers (NHS trusts, a voluntary agency, a local authority social care provider), at three main research sites. IY was chosen as it is the most widely disseminated parenting programme in England with several RCTs demonstrating its effectiveness, based on a thorough curriculum delivered with a strong emphasis on collaboration with parents and fidelity to the model. Phase 1 of PPC was quantitative and aimed to identify key characteristics of parents and children that would predict differential responses to the IY programme. This paper focuses on findings from Phase 2, the qualitative study, comprising interviews with a purposively sampled subset of Phase 1 parents. The PPC project gained regulatory approval from the London Hampstead Research Ethics Committee (N-434-525) and Health Research Authority.

### Data collection

Data were collected over three time points in Phase 1 (at baseline before the IY programme, then three- and six- months after the IY), with Phase 2 qualitative interviews conducted after the third time point for those who completed the IY programme. Parents had been referred to the parenting programme predominantly via children’s services and schools due to their child’s perceived behavioural issues, although there were some self-referrals. For the participants who had not attended or completed IY, data collection points corresponded to similar timescales. The qualitative study therefore aimed to improve understanding of how programmes led to good outcomes for some parents, and poor outcomes for others. Sampling also aimed to encompass variability in parent relationship status (single versus living with partner) and child age and gender.

In total, 139 parents completed Phase 1 baseline data collection with 120 completing data collection at the third time point. Purposive sampling to Phase 2 qualitative study was informed by Phase 1 data, and principally sought to encompass diversity in child behaviour change following the IY programme, based on the quantitative data collected, and parental uptake and ongoing engagement with the IY programme. Consequently, 53 parents were invited to participate in a Phase 2 qualitative interview and 43 (81%) were interviewed; the remaining 10 parents either could not be contacted to arrange an interview after several attempts (*N* = 9), or declined to be interviewed (*N* = 1). Due to a technical problem with audio-recording one interview, 42 interviews were transcribed and included in the analyses. In terms of our principal purposive sampling criteria outlined in the previous paragraph, the distribution of the 42 parents was as follows:

- Group 1 parents (*N* = 14) attended 4 or more IY group sessions and reported improved child behaviour after the IY programme. This was defined as a pre-post change in the Parental Account of Children’s Symptoms scores (PACS [21];) scores of 0.4 SD or greater at the 6 months post baseline follow up. PACS scores were derived from Phase 1 standardised interviews with parents about their child’s behaviour problems “as seen at home” ([21], 761) which were conducted by trained research assistants who rated parents’ responses. It comprises 44 items assessing hyperactivity and defiance.
- Group 2 parents (*N* = 18) attended 4 or more IY group sessions and reported no change or worsening of child behaviour after the IY programme, defined as a pre-post change in PACS score of 0.2 SD or greater, at the 6 months post baseline follow up
- Group 3 parents (*N* = 10) were either: considered for IY but a referral was not taken forward; referred but did not attend IY parenting programme or; dropped out after 3 or fewer sessions. These parents were sampled irrespective of change in PACS scores

Other participant and child characteristics are shown in Table 1. At the time of the interview, the mean age of the children for whom the parents were referred to the parenting group was 6.7 years (range 4–10 years).

**Table 1:** Parent and child characteristics

	N (%)
Parent relationship to child	
Mother	36 (86)
Father	2 (5)
Other carer	4 (9)
Parent relationship status	
Single	18 (43)
Partner	24 (57)
Site	
A	15 (36)
B	13 (31)
C	14 (33)
Child gender	
Male	27 (64)
Female	15 (36)

Phase 1 researchers initially invited parents for interview and provided those interested with a qualitative study information leaflet. Qualitative study researchers usually contacted parents a week later to explain the study further, address questions and arrange interviews. All interviews were conducted face-to-face after researchers had explained their independence from the service delivering the IY programme, the voluntary nature of the study, steps to ensure anonymity and obtained signed consent. Forty semi-structured interviews were conducted by KM and SP, who were experienced qualitative researchers; for logistical reasons, two interviews were conducted by another PPC researcher. Two researchers were usually present at each interview; this was checked with the participants prior to the interview to ensure they felt comfortable. One researcher led the interview, while the other was able to ask questions about issues that could have otherwise been missed. Having two researchers present helped avoid parents being distracted by their children during interviews, as one researcher could play with children as needed. Every participant received a £200 shopping voucher to compensate for their time and any inconvenience caused by participating in the study.

All interviews were conversational and informed by a topic guide. The topic guide was developed by BY and KM based on the previous qualitative literature on parents’ experiences of parenting programmes and adjusted as interviewing progressed to explore previously unanticipated issues and ensure the suitability of questions for parents who had different degrees of engagement with IY or no engagement. Questions followed the parents’ journey from life before IY or PPC, their referral to and experience of IY, perceptions or previous experiences of parenting programmes, what strategies or ‘tools’ they had learned from these and how they had practised the tools with their children, experiences of facilitators and support from the group, suggestions for improving parenting programmes, and what life was like since IY or joining the PPC project. Most interviews were conducted within 2–3 weeks of first contact by the qualitative study team. All were scheduled according to parent convenience and at a place of their choosing, mostly participants’ homes, with five interviews taking place in a café or NHS Trust premises. Interviews lasted between 16 and 94 min (mean = 47) with shorter interviews tending to be with participants from Group 3. Data saturation was reached with Group 1 first, so sampling and recruitment then focused on Groups 2 and 3 until saturation was also reached with these.

### Data analysis

Transcripts of all audio-recorded interviews were checked for accuracy and then anonymised. KM (who has significant experience in qualitative analysis) led the analysis, reading transcripts multiple times and drawing on inductive thematic approaches [4]. The descriptive analysis was framed within a “first-person” perspective ([5], 7) to privilege the accounts of parents, given the ‘outsider’ status of the analysis team. While several team members were parents, none had first-hand experience of the stigma associated with having a child with conduct disorder, or of being referred to a parenting programme, so we felt this framing was appropriate to avoid unwarranted assumptions about parent accounts. Comparison between and within the different participant transcripts ensured similarities and differences in perception and experience were captured. Multiple transcripts were periodically reviewed and discussed by PPC investigators and researchers (KM, MD, JH, EK, SP, RS, BY), thereby bringing different disciplinary, clinical, topic and methodological perspectives to help ‘test’ and refine the analysis. KM checked the analysis with two senior PPC investigators (BY and EK).

As noted above, we use ‘parent’ to refer to all participants in a caring role, and to maintain confidentiality where the carer was not the biological parent. All parents and their children were given a pseudonym. The first time each parent is quoted we refer to them by their pseudonym and their study group number; for example, Lily-1 or Chloe-2.2. Otherwise just the pseudonym is used. Children are only referred to by pseudonym.

## Results

Before describing parents’ narratives in detail, we think it is important to note several important contextual points. Parents’ indicated that service providers were implementing IY in ways that deviated from the specifications of programme developers, and some of these deviations were marked. For example, it was rare for parents to report that service providers had assessed their children before referring parents to the programme. Moreover, several parents reported that they were offered fewer than the recommended 14 sessions, that some sessions were attended by more than the stipulated number of parents, and that contact with facilitators outside of sessions was sometimes absent. We note that many parents spoke in interviews of their experience of attending other parenting programmes, including IY programmes they had attended months or years before the PPC project; Group 3 parents often used previous attendance as a reason behind drop-out or non-attendance. We further note that as interviews and analysis progressed, it gradually became clear that Group 2 was made up of two sub-groups. Sub-group 2.1 comprised 15 parents who, despite the absence of quantitative improvement in their child’s behaviour according to the PACS scores, reported positive perceptions of the parenting programme. During the qualitative interviews, these parents spoke about their belief that their ability to manage their child’s behaviour had improved, which they felt had in turn helped their child. Sub-group 2.2 comprised three parents who did not positively perceive the programme and, consistent with their PACS scores, did not see improvements in their child’s behaviour.

Our analysis identified three themes related to personalisation, engagement, and effectiveness:

1. A personalised approach needs to include the child
2. A supportive school matters
3. The programme needs to feel personal

### A personalised approach needs to include the child

As implemented IY, and other group parenting programmes, do not generally directly include the child in the sessions, although IY programmes that do include children are available [26]. Reflecting this very few of the parents we interviewed described programmes that had directly included their child. Also reflecting the way service providers had chosen to implement IY, as noted above, many parents described being sent to IY without their child undergoing any assessments.

Several parents believed that the absence of any assessment of their child and the child’s exclusion from the programme detracted from the parenting programmes. They felt that programmes needed to look beyond the parent and provide assessment and intervention that directly included the child. No such support was offered by the services running the IY programmes, but it had sometimes been offered by other services. For example, following an assessment outside the service offering the IY programme, Caroline-2.1’s daughter had started to take medication for ADHD with some “psychotic tendencies”. While Caroline had enjoyed IY, she emphasised the tangible difference the assessment and resulting medicinal treatment had made to her daughter’s behaviour: “I did notice a difference in her but you could tell when they wore off …. But it did, it did help for four hours …. Four hours is a good chunk of time” (Caroline-2.1). Further, Anna-3 and Clara-3 spoke highly of their experiences of other parenting programmes which had directly included their daughters and which offered one-on-one interaction with a facilitator. Both mothers recalled how the sessions helped as the facilitator could pinpoint concrete ways to adapt their behaviour with their child on a moment-by-moment basis:

And then she [the facilitator] says to me ‘we will spend ten minutes playing’ so she will be watching me, how we play, how we discuss and then she will explain me what I did wrong! [laughs] … she says to me [that] she will teach me how to change my language, how to use some words, what not to say, what to say, and, yeah, it’s good. (Anna-3)

[The facilitator would] stand behind me … she’d keep guiding me what to do …. Because she was there it sort of gives you that extra strength to sort of go ‘right OK’ and then she’ll sort of signal and she’d sort of whisper in my ear … what to do and what to say …. it was though she wasn’t there, but she was. (Clara-3)

However, the desire to include their child in a parenting programme was voiced most strongly by parents who did not think their child’s behaviour improved after IY or other parenting programmes. For example, both Chloe-2.2 and her husband felt that none of the strategies or tools they were taught in IY were effective with their son. Chloe described the facilitators as insisting she persist with the tools, which Chloe attributed to an assumption by the facilitators that she was not implementing the tools correctly. Chloe pointed to the flaw in this: “Well, how do you [the facilitators] know that? You’ve never met him” (Chloe-2.2). Chloe’s husband likened the facilitators not including their son in the intervention to a mechanic not seeing the car before trying to fix it:

I’ve got a problem with the car. I’ve got a mechanic coming out. The first thing the mechanic does, is look at the things to sort of try and work out the problem. So, they may well be wasting their time because if they came out and saw the child, they could go, ‘alright, okay, I think he can do with some help but … we don’t think we’re quite the right people’. (Chloe-2.2’s husband)

These parents often perceived assessment and diagnosis to be a gateway to receiving appropriate support for their child. They had already experienced considerable negative judgement from others regarding their child’s behaviour, and far from feeling that diagnosis would exacerbate this, they regarded it as key to understanding their child and making sure he or she had the help they needed. Felicity-3 knew her son wasn’t ‘bad’, but she also knew he needed more support than she could give, or that services had so far, offered him. Her focus on gaining this support overrode other concerns:

It’s more to the fact that so that people don’t look at him like he’s a bad child because he isn’t a bad child …. He has his moments, yes but every child does. But he has his moments more often than other children. And it’s more so that he can get the support that he needs …. Rather than me, I couldn’t give like a damn about me. As long as he’s getting the support. (Felicity-3)

Louise-1’s experience of IY had, at least in part, given her what Felicity was still looking for. IY had helped Louise to:

Realise that there’s more to what’s going on with [Marianne] … than what we were doing, if that makes sense, as parents. There was an underlying issue that we need to resolve [laughs] – rather than it just being us that aren’t necessarily doing the right things. (Louise-1)

While her daughter, Marianne, had not received an assessment or diagnosis prior to being offered the IY programme, and Louise saw ways that Marianne’s behaviour had improved as a result of using the strategies from the programme, Louise also saw that Marianne had ‘an underlying issue’ that needed further assessment. Louise spoke about needing to wait until Marianne turned the ‘right’ age before this assessment could be carried out. She also pointed to the work she had done to make sure the assessment was carried out promptly, including keeping a systematic list of Marianne’s symptoms ever since she had begun displaying them:

Well, when she turned seven, I took her to the doctor with the list of symptoms and literally they said ‘normally I’d send you away for six months and ask you to monitor it and come back’. He said, ‘but I can see you’ve dealt with this for seven years and you need some help’. I said ‘yeah’ [laughs] …. And we sent that off to a paediatrician and they came back quite quickly. So now, we’re just waiting to find out what the next steps are to help her. (Louise-1)

Nadia-2.1 gave an example of the transformation in her son’s wellbeing after he had been assessed and diagnosed with speech difficulties. Prior to the diagnosis, Nadia felt the staff at her son’s first school had blamed her for his difficulties. Now that he was receiving speech therapy at his new school, he was better able to socialise: “We’ve been to birthday parties …. play dates continuously” (Nadia-2.1). Nadia believed schools needed to listen to parents more rather than assume a child’s problems was all the fault of parents: “Give the benefit of the doubt. Listen …. if parents can be listened [to] more, you know?”

However, as the reports of some parents demonstrated, assessment did not always provide a gateway to appropriate support for a child. Adam-3’s son had been assessed but nothing conclusive had been found – Finn was ‘on the spectrum’ for many things: “[Finn] now has attachment disorder, traumatic disorder …. He’s on a spectrum for loads of things, Tourette’s, autistic.... Loads of stuff”. Adam and his son had been involved with CAMHS for several years with nothing ever seeming to improve. Adam wondered whether CAMHS was simply prevaricating as a diagnosis would mean the service would have to act. Violet-2.1 found herself in a similar situation with her child, where assessments had been conducted but nothing was conclusive. She spoke about several diagnostic possibilities, but with little sense of direction regarding treatment or support for her child:

I don’t know if they’re planning on giving him a diagnosis …. for anything. The ASD is a definite no-no …. the attachment syndrome... it’s looking like that’s what it is … he’s come from care … But how they’re dealing with it, I don’t know. I don’t know what they’re doing about it. I don’t know whether there’s a... a road to go down to see if there’s a diagnosis or that’s what it actually is or?. (Violet-2.1)

### A supportive school matters

Given the length of time children spend at school, the effectiveness of strategies parents learned at the parenting programme could be limited if the child was not also supported at school. Some parents reported excellent support from their schools for their child, and through this for the parents themselves. Yet support from schools often depended on a child having been formally diagnosed and an Education, Health, and Care Plan (EHCP) being in place. For example, some schools had separate spaces or groups that allowed children to have a quieter time during their lunchbreak. Elodie-2.1’s experience demonstrated the difference a good Special Education Needs Co-Ordinator (SENCO) could make to a child’s behaviour. Elodie felt that she had not been taken seriously when she first raised concerns about her son’s behaviour in nursery, and so no support had been provided for Caleb. However, when a new SENCO arrived in the school, she helped Elodie put an EHCP into place which provided lasting benefits:

But then a new SENCO came in when we had the bad year and she was fantastic. I mean she helped me, in a year we got the EHCP, she got me all the help I could get, she got me into CAMHS, you know, so having a good SENCO has made a huge difference …. She left now to be a deputy head and I felt quite sad. (Elodie-2.1)

Other parents had more problematic experiences, finding it hard to ensure adequate support for their child in school and making sure responses to their child’s behaviours were consistent across home and school. For his first year of school, Charlotte-2.1 felt her son, James, had received the support he needed, including speech therapy and focused classroom roles, even though he had yet to be formally diagnosed. James was about to move up a grade and the school were not planning to continue this additional support and Charlotte-2.1 worried about this: “He isn’t – I hate the word normal – but you know what I mean?... he does have these issues, so yeah, you have to do certain things with him to get him to feel comfortable, get him.... to join in more rather than just saying ‘You just do this’”. As a result, Charlotte was now pushing for an assessment and diagnosis so James could access the help he needed – and the school would have the funding in place to provide it – but it was a slow and seemingly frustrating process:

[I] spoke to the SENCO the other day, they haven’t looked at him or done anything with him for some time …. There’s not much we can do! But again they can’t get funding to put something into place for him.... until he gets the diagnosis. (Charlotte-2.1)

Poppy felt that the school’s knowledge of her past interactions with social services meant her son’s school teachers always assumed the worst. Poppy’s son, Jack, was 5 years old at the time of the interview and she had been involved with CAMHS since his birth. She described an incident when a teacher had rung social services to report concerns about Jack eating breakfast at school, despite this being a routine that had been agreed with another teacher given the difficulties Poppy faced getting him ready in the morning: “I was [saying] … would you have rung social services on the person or a normal parent who has never had social services before – just because you know my background?” After a few other incidents where Poppy felt a teacher’s neglect had harmed Jack, she initiated a ‘communication book’, whereby she and the teacher would note details of Jack’s behaviours each day so that they each knew how Jack was doing and could adjust how they interacted with him accordingly. Even with this in place, Poppy felt she was communicating more with the school than they reciprocated. She described having to advocate for Jack every step of the way and her fear of being blamed for not doing enough: “I feel like if I’m not fighting and trying to get [son] what he’s entitled to or get help, then they’re going to come to me and say ‘well, you didn’t do this … or, you know, ‘you’ve got to discipline him’”. Here, Poppy demonstrated the precariousness that was shared by other parents who were users of social services – they had to constantly be seen to be ‘active’ or ‘better’, even when they were struggling and feeling little or no support was being offered.

Adam had similar frustrations with his son, Finn’s, former school. Adam felt the staff at Finn’s school ignored his suggestions on how to better support Finn, while Finn’s behaviour deteriorated to the point of him being excluded from school. Adam recounted what happened when Finn’s grandfather suddenly passed away during a holiday, and Finn returned to school grieving:

And he was a major part in [Finn]‘s life …. So then [Finn] went back to school, still... well we were really upset, and school didn’t help out there …. When he was having his outbursts, like they would just put him into this little room and … he’d was being pinned down to the floor like …. not comforting him... [or] talking about his feelings. (Adam-3)

Since being excluded, Finn was attending another school for 1 hour a day which Adam believed was not meeting his behavioural, emotional, or educational needs, but it was all that was available. As a result, Adam was working to get his son into a school that specialised in educating and supporting students with behavioural needs.

### Making the programme feel personal

Even though IY was a group-based programme, parents described ways in which it could be made to feel personal. Making the programme feel personal could involve practical steps such as making sure the group was at a convenient time and place, but it also included the ways that facilitators responded to parents as individuals and the particular needs or difficulties they brought to the groups. This influenced attendance, and whether parents felt they or their child benefited from the programme.

Josie-2.1 described how the efforts that the IY facilitators made to support parents in voicing their experiences and then incorporating these as part of the learning. This helped Josie feel the strategies and tools were more relevant:

I was happy with the support that I got from the group because I was able to go back and feedback and also I was able to hear what …. other people have [done] and how they resolved it. And how they [the facilitators] used what we were taught in the group to resolve it. (Josie-2.1)

However, other parents described the programme as not feeling personal. Some parents believed they had little choice over attending a programme. For several of these parents, the feeling that the programme was not personal stemmed from the point of referral. However, the feeling that the programme was impersonal was also often related to travel times and distances, where even if a parent felt IY or another parenting programme would have been helpful, it was physically impossible for them to get to the venue where the group was held. Indeed, Olivia-1 suggested online programmes could mitigate issues around travel and time and, consequently, help make a programme feel more personalised for the parent: “like it [the group] is the commitment. It’s like having to commit to an extra thing.... well parents who have lots of kids … juggling and juggling and juggling and trying to squeeze it in for two and a half hours” (Olivia-1).

Participants who had a positive experience of the IY programme spoke about the groups and sessions being a ‘safe place’, which made being there feel more like a personal choice, even if the parent had initially felt compelled to join a programme rather than having freely chosen to attend. The language such parents used to speak about the programme was that of belonging and safety. Jac-1 found that being able to talk openly about her difficulties with her child made the group feel “like family. We were like family”. Further, given that their children were not usually seen in a positive light by others, the programme allowed parents to tell stories of their child’s achievements, not just of their challenges: “we had somewhere to go and brag about our children. Good or bad. So, that was nice, and everybody there was listening” (Lily-1). Indeed, Vivienne-2.1 had already tried IY previously and dropped out, as the facilitator in the previous group had not been able to foster cohesiveness among parents after one revealed acts of physical aggression: “one of the women said, ‘oh, yeah, I punch my child’ and I’m there going, ‘what?’ And I, because I said, I don’t believe in child punishment … none of [the other parents] actually spoke to me” (Vivienne-2.1). In contrast, Vivienne had found her latest programme was a safe space because of the group connection: “the right people.... once you fit, you feel that you fit.... That group, everyone was just, we all bounced off each other, the questions were answered” (Vivienne-2.1).

However, when an IY programme did not feel like a safe space, attending could feel like a tick-box exercise rather than a personal choice: “Yeah, I felt like I had to tick that off. Like everything that they threw at me, near enough, I felt like I had to tick off” (Claire-3). After feeling like she had no choice but to attend IY, Lucy-2.2 felt the facilitators had betrayed her by seeking, via her social workers, information about her pre-existing relationship with CAMHS. Lucy remarked that this had led to “some funniness with the facilitators” (Lucy-2.2). She added that the facilitators had made assumptions about her situation from the beginning, and undermined her during the sessions:

Towards the end I didn’t [feel safe] because the other thing is, we have social workers with our – on our case …. I started [to talk] about my issues and not being able to get things done in the house and stuff like that. And they [the facilitators] went like, [puts on a bored voice] ‘Well, you just kind of have to, don’t you?’ …. things that I’ve said, [the facilitators have] said, ‘Oh yeah, we know about that.’ So that means that the social workers had already spoken to them. (Lucy-2.2)

While information may have been shared for safeguarding reasons with the intent to help Lucy and her family, being left out of this process meant Lucy thought the worst. Indeed, Lucy spoke about becoming distressed and crying during, and at the end of, some sessions and not receiving any offers of support or comfort from the facilitators.

Clara ended up feeling similarly unsafe despite having chosen to do IY. This was her second time on the programme. The first time she completed the programme was shortly after her partner’s suicide. She wanted to do IY a second time to refresh her memory of the strategies and tools. Clara related her dropping out of IY to an incident that took place just before one of the sessions, when her youngest child, a toddler, had refused to go into the childcare area. The childcare workers did not offer to help settle her child, rather they complained that Clara had been too rough when bringing her toddler into the crèche. The childcare workers later recanted but Clara no longer felt “safe” in the programme:

It was afterwards when it got reported back to me like a few weeks later from one of the facilitators ‘Oh the crèche have realised … what they should have done is maybe come and help you, come and encourage [daughter] to come into there’ …. That’s exactly my point … and I said ‘Well I’m glad that I was able to help them! You know I’m glad that I was able to facilitate them learning that!’ [laughs] …. but then you’re sort of nervous to …. say anything back. (Clara-3)

While not every strategy taught by the parenting group was appropriate or effective for everyone, how group facilitators handled this could make the group both a safer and more relevant place for parents, or a more judgmental and irrelevant place. Indeed, Claire and Zara-3 had dropped out of IY in part because they felt what they had been taught was not relevant to their families. Similarly, Chloe felt that the group leaders delivering the IY programme had focused on strategies that did not work for her son. As previously noted, Chloe felt the facilitators essentially assumed that she and her husband were not implementing the strategies properly. Chloe was left alone to wonder how to interact with a child who never seemed to respond positively to her:

When people came out [to do assessments], he’s quite happy to play with me. But on our own, he won’t. He literally won’t. And he’ll go out of his way to avoid it. Um, I don’t mean that as like he’ll just go out of his way to avoid it. I mean, he’ll purposely be naughty. And it’s difficult. And they’re just, ‘oh be persistent’. I’m like, ‘how am I meant to be persistent if he doesn’t want me to do it?’(Chloe-2.2)

## Discussion

In this study we examined the narratives of four different parent groups: Group 1 parents whose positive reports of the IY programme and improved child behaviour mirrored the PACS quantitative measure; Group 2.1 parents whose positive accounts of the IY programme and improved child behaviour during their interviews did not mirror the quantitative PACS scores; Group 2.2 parents whose negative experience of the parenting programmes and no improvements in their child’s behaviour was consistent with their PACS scores; and, Group 3 parents who had dropped out, did not attend, or were considered for, but not referred to, IY. We had originally envisaged examining the different experiences of Groups 1, 2, and 3, and did not anticipate the emergence of the split in Group 2 from the analysis. Nevertheless, such divergence of quantitative and qualitative data in mixed-methods studies is not uncommon [17] and exploring these divergences can offer important insights. Parents in Group 2.1 often described feeling more confident in dealing with the problems they still faced with their child. In contrast parents in Group 2.2 described negative experiences of the programme and remained hopeless about their situation. They had tried something else to help their child, yet the difficulties remained.

Attride-Stirling et al. [2] described similar distinctions between programme ‘completers’ and ‘non-completers’: “… completers did not feel that the problems were vanishing before their eyes; but they focused more on *how* they were changing, rather than how much. Non-completers, on the other hand, conveyed a sense that the battle had already been lost” (2004, 355). In our study, both sets of Group 2 parents attended sufficient sessions to be categorised as having completed IY, yet Group 2.1 parents spoke about feeling personally engaged in the programme, a sense of group belonging, and the facilitators helping them adapt tools as relevant to their needs. We propose that this positive experience helped Group 2.1 to hope that their child’s behaviour could change because they were using IY tools and strategies adapted for their family. In contrast, Group 2.2 parents reported feeling betrayed by the facilitators, who parents perceived did not take their concerns seriously, and who, rather than offering suggestions for adapting the tools, left parents feeling blamed when tools were ineffective. This experience confirmed to Group 2.2 that parenting programmes were not relevant for their child and family. Parents’ narratives also demonstrated that even when parents from both Groups 2.1 and 2.2 felt they had been made to attend a parenting programme via a service referral, only parents in Group 2.1 reported feeling that was group was appropriate for and accepting of them. We note that while Group 3 also spoke about perceptual barriers, such as feeling forced into a programme, and that structural barriers [29], such as travel times and distances, also constrained their ability to attend the IY programme.

Understandably, personalisation in the context of a parenting programme focusses attention on the parent, as they are the ones directly involved in the intervention. However, a number of parents wanted their children to be included in the programme sessions. This was especially prominent in the narratives of parents whose PACS scores remained high, indicating their child’s behaviour was consistently concerning. Some had undertaken other parenting programmes that had directly involved their child and these parents were overwhelmingly positive about such programmes, even if subsequent challenges in their lives and the lives of their children had triggered a subsequent referral to a parenting programme. The need to include children was also evidenced by the powerful thread through many parents’ arguments of wanting assessment and diagnosis. Parents struggled to engage with IY and other parenting programmes when they felt their parenting was not the only issue and that their child also needed assessment and specialist support, often for ADHD or ASD. Support from schools often required an assessment and diagnosis too, and also influenced a parent’s ability to engage with IY and whether they described quantitative or qualitative improvement in their child’s behaviour after the programme. Most studies to date have either examined a parenting programme without looking at external influences like school [28], or have focussed specifically on how parenting programme can be implemented in a school environment [19]. Findings from the current study suggest that a focus on the home environment alone may not be enough, and that the school environment can also be important in improving a child’s behaviour. Indeed, co-ordination across both environments may be needed for any parenting programme to be effective for children with complex diagnoses and support needs [19].

Parents’ narratives suggest that current service delivery of IY parent programmes is not always consistent with existing national guidelines for children with conduct problems in the UK. Indeed, the perspectives of parents in this study align well with these guidelines, which recommend that children’s difficulties be assessed before services are offered, that individual programmes are provided for parents who cannot attend groups and that programmes be offered that include children directly (NICE [16]). As viewed through these parents’ reports, the service delivery of the programmes is also inconsistent with the stipulation of programme developers that programme delivery be tailored to meet individual needs [26], including joint sessions with parents and children where parents need direct support in using the programme tools and strategies. Parents’ experiences also indicate that personalisation within a parenting programme was not always about individualisation but about the quality of the facilitation. For example, by taking the time to talk to a parent about adapting a tool or strategy, facilitators could dismantle perceptual barriers and help make an ostensibly standardised group experience feel more personal for a parent. Consequently, parents were able to engage with the IY programme. In contrast, when facilitators did not address perceptual barriers, or in some cases reinforced these, parents became less engaged, some to the point of dropping out of IY.

In this way, there is a need for parenting programme facilitators, and those referring parents to programmes, to understand the different motivations parents can have before and during a programme. Engaging with parents’ different motivations, offers a way of supporting parents to experience a programme positively, and this positivity could extend to how they perceived their child. The findings also raise wider questions about whether personalisation provides a useful way of conceptualising the support parents need and what might be lost as well as gained from personalisation. The social connectedness that parents experienced when they thrived in a group-based programme environment is important here. A shift away from group-based programmes as a way of directly including children or when a group environment is anticipated not to be suitable for a parent, would remove the opportunity for such social connectedness. The drawbacks of this need to be considered in developing personalised programmes.

Practical elements were also important to parents, for example, ensuring a location was accessible was important in enabling parents to attend. The material resourcing of parenting programmes also needs to be considered. An IY programme in a previous study provided parents with travel expenses or assistance, refreshments, prizes, a book, certificates, and completion gift ([28], 9). Apart from childcare, these types of benefits were not often offered to the parents in the current study. The absence of support for travel, or availability of programmes close to parents certainly limited the accessibly of the IY programme, while and long and complicated journeys made engagement more difficult for parents whose frame of mind was then hardly conductive to learning. In this way, accessibility needs to be considered as a part of personalisation because what is just an inconvenience to one person can be insurmountable to another [2]. Overcoming structural barriers to attendance is recommended as standard practice by IY programme developers [25] and is a requirement of national guidelines in the UK (NICE, [16]).

This study has some limitations. Many of the participants had attended several other parenting programmes in the past, including IY. This could have influenced parents’ motivation and engagement with IY in the present study. We were unable to fully explore some of these experiences in the interviews, and there is a need for further exploration around programme fatigue and its impact on future programme effectiveness. The narratives of some parents pointed to a marked disconnect between how IY was intended to be run and how it was delivered, and to problems with the quality of facilitation. We cannot rule out the possibility that our interpretations might been different had IY been delivered in the ways that were aligned with the specifications of the programme developers and standards in national guidelines. In addition, we were not able to fully explore the impact socio-economic status might have had on parents’ experiences of a parenting programme.

The PPC Study used mixed methods which Weeland et al. [28] argue gives a more holistic understanding of the parent and child. While quantitative data was collected at three time points, longer-term follow-up via qualitative interviews at all time points would have offered insights on the sustainability of improvements. Relying on parent self-report data for the PACS may be less robust than using sources such as a teachers ([20], 101), but teacher reports were not feasible given our resource constraints. Further, while more women participated than men, and more male children were referred than female children, this is also representative of who attends parenting programmes and that boys are more likely to be found to demonstrate conduct problems than girls. The inclusion of children’s accounts needs to also be considered in future studies, especially where they are directly involved in the programmes.

## Conclusions

This study examined how the reach of parenting programmes could be extended, based on the experiences of parents referred to such a programme. The findings indicate the importance of dismantling perceptual and structural barriers to participation and engagement. Such dismantling will not only require material resources, but staff who can connect with parents and respond to their individual needs and those of their child. Further work to examine how programmes can prevent a disconnect between the intended delivery of parenting programmes and the ways that services actually deliver the programmes in practice would be useful. Our findings point to a need for personalised programmes to assess children before parents are referred to parenting programmes. The findings also point to the value of working collaboratively with parents and children to better identify who would benefit most from group support or one-to-one support and how this may change depending on circumstances. Liaising with schools to ensure they support the child in line with what the parent is learning will also be important. Parents’ narratives have enabled us identify which elements to include in a personalised parenting programme. Detailed development and evaluation of such a programme is now required to discover whether personalisation can extend the benefits of parenting programmes to a wider range of parents and children.

## Data Availability

The dataset generated and analysed during the current study are not publicly available due to potential for breach of anonymity, but are available from the corresponding author on reasonable request.

## Abbreviations

ADHD: Attention Deficit Hyperactivity Disorder
ASD: Autism Spectrum Disorder
CAMHS: Child and Adolescent Mental Health Services
EHCP: Education, Health, and Care Plan
IY: Incredible Years
PACS: Parental Account of Children’s Symptoms scores
PPC: Personalised Programmes for Children
SENCO: Special Education Needs Co-Ordinator.
